# A case of penta X syndrome caused by nondisjunction in maternal meiosis 1 and 2

**DOI:** 10.1002/ccr3.1004

**Published:** 2017-06-01

**Authors:** Sara Markholt, Jesper Graakjaer, Signe Bødker Thim, Bente Høst, Anne‐Bine Skytte

**Affiliations:** ^1^ Department of Clinical GeneticsAarhus University HospitalAarhusDenmark; ^2^ Department of Clinical GeneticsLillebaelt HospitalVejleDenmark; ^3^ Department of PediatricsAarhus University HospitalAarhusDenmark

**Keywords:** Dilated intestines, genotyping, hypoplasia of the corpus callosum, microarray, nondisjunction, Penta X syndrome, Pentasomy X, short femora, STR marker

## Abstract

The prenatal abnormalities in patients with penta X syndrome appear late in pregnancy and are nonspecific. In contrast, the postnatal phenotype is well described although new findings are still revealed. Penta X syndrome is a result of successive nondisjunctions of the X chromosomes in both maternal meiotic divisions.

## Introduction

Penta X syndrome is a rare sex chromosomal anomaly. The first case was described in 1963 1, and since then, only ~26 postnatal and five prenatal cases have been reported. The pathogenesis of penta X syndrome has been hypothesized to be successive nondisjunctions of the X chromosomes in both maternal meiotic divisions 2, 3, 4. Our study provides a detailed pre‐ and postnatal clinical evaluation and is the first study to show, on the basis of genotyping of SNP‐array data, that successive nondisjunctions of the X chromosomes in both maternal meiotic divisions can be the cause of penta X syndrome.

## Clinical Evaluation

The girl was the first child of nonconsanguineous parents. The mother was aged 26 years, and the father was aged 32 years. Conception was natural, and the family history was unremarkable.

### Prenatal phenotype

The pregnancy was uneventful until gestational age (GA) week 28. No abnormalities were detected at the ultrasonographic examination at GA week 13+1 and at the prenatal anomaly scan at GA week 20. At GA week 28, polyhydramnios was detected during a routine midwife appointment. Regular fetal ultrasonographic examinations from GA week 28 until delivery revealed polyhydramnios, dilated intestines, IUGR with −23.1% to −30.7% weight deviation, and short femora (−2.8 SD). Because of suspected brain sparing, the birth was initiated at GA week 38+3.

### Postnatal phenotype

The newborn girl had a birthweight of 2484 g, a length of 48 cm, and a head circumference of 34 cm. She had hypertelorism, abnormal configuration of the ears, cleft palate (in the soft palate), bilateral single transverse palmar creases, bilateral clinodactyly of the fifth finger, short lower limbs compared to the rest of the body, mild hypotonia, and a shrill animal‐like cry. Echocardiography showed mild stenosis of the right pulmonary artery and mild coarctation of aorta, none of which were thought to have clinical significance. Ultrasonography of the brain showed hypoplasia of the corpus callosum, and the brain parenchyma appeared slightly more echogenic than normal. Furthermore, a few subependymal pseudocysts were observed on the right side and a single subependymal pseudocyst was observed on the left side. All findings by ultrasonography of the brain were of unknown clinical significance. Based on the clinical impression, there was initially no need for further follow‐up. No structural eye abnormalities were found at examination by ophthalmologist. Ultrasonography of the urinary tract did not reveal any abnormalities. The girl was discharged when she was 17 days old still requiring enteral tube feeding as a supplement to bottle feeding because of feeding difficulties and failure to thrive. Close follow‐up was planned. The patient's clinical features are summarized in Table 1.

**Table 1:** Clinical findings in the patient

1st trimester pregnancy	Normal ultrasonographic examination with nuchal translucency = 1.8 mm (<95th percentile)
2nd and 3rd trimester pregnancy	IUGR
	Polyhydramnios
	Dilated intestines^a^
	Short femora^a^
	Brain sparing
Neonatal	Low birthweight (2484 g)
	Hypertelorism
	Abnormal shape of the ears
	Cleft palate
	Bilateral single transverse palmar creases
	Bilateral clinodactyly
	Short lower limbs compared to the rest of the body^a^
	Mild hypotonia
	A shrill animal‐like cry^a^
	Mild stenosis of the right pulmonary artery and mild coarctation of aorta
	Hypoplasia of the corpus callosum^a^
	Subependymal pseudocysts^a^
	Failure to thrive

a Not previously reported.

## Laboratory Findings

Shortly after birth, the midwife suspected a syndrome, and the girl was therefore examined by a physician at the maternity ward. The physician ordered chromosome analysis and aneuploidy screening without having any specific syndrome in mind.

### Chromosome analysis

Standard chromosome analysis with Q‐banding was performed on cultured peripheral blood lymphocytes from the patient and revealed the karyotype 49,XXXXX in all 10 examined metaphases.

### Aneuploidy screening

Standard aneuploidy screening QF‐PCR analysis for chromosomes 13, 18, and 21 as well as the sex chromosomes was performed on DNA extracted from a peripheral blood sample from the patient. The results showed a triallelic Pentasomic pattern in 2 of 3 markers on the X chromosome. The third marker was borderline within normal range.

### SNP‐array and genotyping analysis

To clarify the pathogenesis of penta X syndrome, SNP‐array analysis (Illumina CytoSNP‐12 v2.1 format; standard protocol) followed by genotyping analysis of the data (Illumina Genomestudio v2011.1 and Microsoft Excel 2010) was performed on DNA extracted from the patient and her parents.

**Figure 1:**
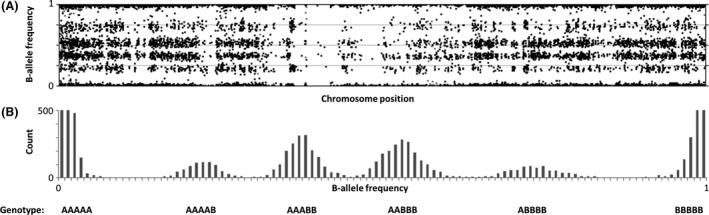
(A) BAF plot illustrating the distribution on the patient's chromosome X. (B) BAF frequency plot illustrating the grouping of BAF. Each BAF group corresponds to a genotype (AAAAA, AAAAB, AAABB, AABBB, ABBBB, BBBBB).

SNP‐array data are given as B‐allele frequencies (BAF) with a value between 0 and 1 representing the observed frequency of single nucleotide polymorphisms (A or B alleles). To perform genotyping analysis, the BAF must be translated into genotype data. The SNP‐array data from the patient showed six different BAF groups for the X chromosome, corresponding to the genotype: AAAAA, AAAAB, AAABB, AABBB, ABBBB, BBBBB (5 X chromosomes; Fig. 1A and B). The observation of six different BAF groups confirmed the diagnosis of penta X syndrome. The SNP‐array data from the mother showed three different BAF groups for the X chromosome, corresponding to the genotype: AA, AB, BB (2 X chromosomes). The SNP‐array data from the father showed two different BAF groups for the X chromosome, corresponding to the genotype: A or B (1 X chromosome). These observations confirmed a normal X chromosome constitution for both parents.

To investigate how many of the patient's X chromosomes that were of paternal origin, we identified SNPs with opposite parental genotypes. Thus, SNPs where the father's genotype was A and the mother's genotype was BB, as well as SNPs where the father's genotype was B and the mother's genotype was AA, were identified. If the father's genotype was A, the number of A in the patient's genotype revealed how many copies of the father's X chromosome the patient had inherited. Our analysis showed that opposite parental genotypes were observed in 822 cases. In 818 of these 822 cases, the patient's genotype showed ABBBB or AAAAB. The result indicates that only one of the patient's X chromosomes was inherited from the father.

Having established the paternal contribution, there were now three possibilities with regard to the origin of the patient's four maternal X chromosomes:

- All 4 X chromosomes originated from the same maternal X chromosome (maternal X chromosome genotype: AAAA, BBBB).
- Both homologue maternal X chromosomes were duplicated once (maternal X chromosome genotype: AABB).
- Both homologue maternal X chromosomes were inherited to the patient, and there was a triplication of one maternal X chromosome (maternal X chromosome genotype: AAAB, ABBB).

Each possibility was investigated separately:

- If all four X chromosomes originated from the same maternal X chromosome, the possible genotypes would be AAAAA, ABBBB, AAAAB, BBBBB. This combination of maternal and paternal X chromosomes would result in four BAF groups and not in six BAF groups as seen in our analysis. Therefore, this pathogenesis could be rejected.
- If the patient's penta X syndrome was caused by a duplication of both homologue maternal X chromosomes, the expected genotype would be as indicated in Table 2, column 6. The expected patient genotype was calculated from the observed genotype of both parents. A comparison between the expected genotype (column 6) and the observed patient genotype (column 3) showed that they were identical in 14979 of 15018 cases. Because of so few discrepancies, we assess that the result indicates that the patient's penta X syndrome was caused by duplication of both homologue maternal X chromosomes.
- If the patient's penta X syndrome was caused by a triplication of one of the maternal X chromosomes, the expected genotype, after subtraction of the father's genotype, would be either AAAB or ABBB in those SNPs where the mother's genotype was AB. When the father's genotype was subtracted from the patient's observed genotype, it resulted in a balanced genotype (AABB) in 3785 of 3812 informative SNPs. Thus, there was not consistency between the observed genotype (AABB) and the expected genotype (AAAB/ABBB). The result indicates that the patient's penta X syndrome was not due to a triplication of one of the maternal X chromosomes. Instead, the result indicates that the patient's penta X syndrome was caused by duplication of both homologue maternal X chromosomes.

In conclusion, our genotyping results show that the patient's penta X syndrome was caused by duplication of both homologue maternal X chromosomes.

**Table 2:** Extraction of the genotype data

Chr	Position	Patient genotype (observed)	Mother genotype (observed)	Father genotype (observed)	Expected patient genotype if both homologue maternal X chromosomes were duplicated
X	2704609	BBBBB	BB	B	BBBBB
X	2710840	AABBB	AB	B	AABBB
X	2711289	AAAAA	AA	A	AAAAA
X	2711429	AABBB	AB	B	AABBB
X	2712661	BBBBB	BB	B	BBBBB
X	2714756	AAAAA	AA	A	AAAAA
X	2719111	BBBBB	BB	B	BBBBB
X	2727310	BBBBB	BB	B	BBBBB
X	2735539	AAABB	AB	A	AAABB
X	2737851	AAABB	AB	A	AAABB
X	2743627	AAAAA	AA	A	AAAAA
X	2743954	AAAAA	AA	A	AAAAA
X	2744765	AAABB	AB	A	AAABB
X	2746558	BBBBB	BB	B	BBBBB
X	2832001	AAAAB	AA	B	AAAAB

### STR‐marker analysis

**Figure 2:**
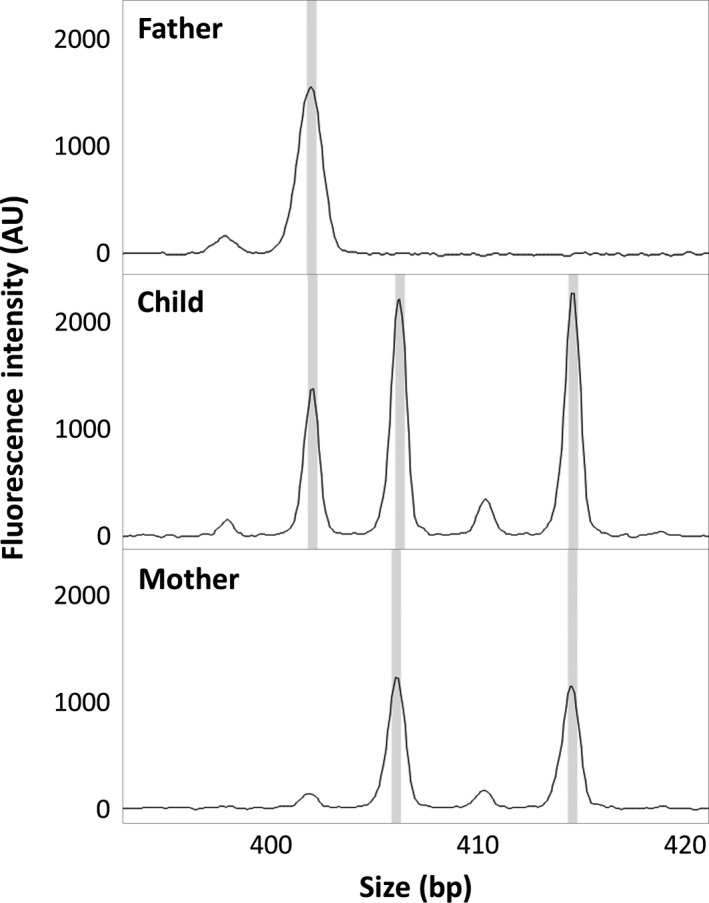
STR‐marker analysis illustrating the triallelic pentasomic pattern observed for the patient's chromosome X.

To confirm the genotyping results, STR‐marker analysis (Elucigene QST*Rplusv2; standard protocol) was performed on DNA extracted from the patient and her parents. The analysis included 11 polymorphic STR‐markers spread on the sex chromosomes and one nonpolymorphic sex chromosome‐specific marker. Comparison of the results of the patient and the parents showed, in two loci with informative distribution of alleles, that one allele was inherited from the father and four alleles were of maternal origin, with two alleles from each of the X chromosomes of the mother (Fig. 2). In five loci, the distribution of the alleles was compatible with the same inheritance pattern. The remaining five loci were noninformative. The STR‐marker analysis could thereby confirm that the patient's penta X syndrome was caused by duplication of both homologue maternal X chromosomes.

## Discussion

The clinical examination of our patient both prenatally and postnatally adds further to the phenotype in individuals with penta X syndrome. Previous findings could be confirmed, and new findings, such as the dilated intestines and the short femora found prenatally as well as the shrill animal‐like cry, short lower limbs, hypoplasia of the corpus callosum, and subependymal pseudocysts found postnatally, are suggested to be part of the phenotype in penta X syndrome 1, 2, 3, 5, 6.

Our genotyping and STR‐marker analysis show that the penta X syndrome of this patient was caused by duplication of both homologue maternal X chromosomes. Our results add further evidence to the hypothesized theory that penta X syndrome is caused by successive nondisjunctions in both the 1st and 2nd maternal meiotic divisions 2, 4, 7, 8.

The mother's age as a predisposing factor has been evaluated in earlier studies. In one study reviewing 23 cases of penta X syndrome, the mean maternal age was 27 years 2. In another study, the maternal age was <35 years in 81% of 21 cases of penta X syndrome 5. These results indicate that there is no correlation between advanced maternal age and increased risk of penta X syndrome. This is also consistent with our case, where the mother was aged 25 years at conception.

As illustrated in our case and in accordance with previously reported cases 5, 7, 9, 10, 11, prenatal diagnosis of penta X syndrome remains a challenge. Prenatal abnormalities are generally detected late in the pregnancy and are nonspecific. This, however, only underlines the importance of a continued characterization of penta X syndrome cases. Our case and previously reported cases have identified new phenotypic features such as corpus callosum hypoplasia and clinodactyly that are possible to detect prenatally. Further characterization and technological advances may aid in early diagnosis of penta X syndrome in the future.

## Authorship

SM: involved in developing the idea, some of the data analysis, drafting the article, critical revision of the article, and final approval of the version to be published. JG: involved in most of the data analysis, critical revision of the article, and final approval of the version to be published. SBT: involved in the clinical examinations, critical revision of the article, and final approval of the version to be published. BH: involved in the clinical examinations, critical revision of the article, and final approval of the version to be published. A‐BS: involved in developing the idea, some of the data analysis, critical revision of the article, and final approval of the version to be published.

## Conflict of Interest

All authors declare no conflict of interest.
